# Influence of own mother's milk and different proportions of formula on intestinal microbiota of very preterm newborns

**DOI:** 10.1371/journal.pone.0217296

**Published:** 2019-05-20

**Authors:** Adriana Zanella, Rita C. Silveira, Luiz F. W. Roesch, Andréa L. Corso, Priscila T. Dobbler, Volker Mai, Renato S. Procianoy

**Affiliations:** 1 Unidade de Neonatologia do Hospital de Clínicas de Porto Alegre, Universidade Federal do Rio Grande do Sul, Porto Alegre, Rio Grande do Sul, Brazil; 2 Centro Interdisciplinar de Pesquisas em Biotecnologia–CIP-Biotec, Campus São Gabriel, Universidade Federal do Pampa, São Gabriel, Rio Grande do Sul, Brazil; 3 Department of Epidemiology, College of Public Health and Health Professions and College of Medicine, Emerging Pathogens Institute, University of Florida, Gainesville, Florida, United States of America; University of Illinois at Urbana-Champaign, UNITED STATES

## Abstract

**Objective:**

To determine the differences in preterm infants’ stool microbiota considering the use of exclusive own mother’s milk and formula in different proportions in the first 28 days of life.

**Methods:**

The study included newborns with GA ≤ 32 weeks divided in 5 group according the feeding regimen: 7 exclusive own mother’s milk, 8 exclusive preterm formula, 16 mixed feeding with >70% own mother’s milk, 16 mixed feeding with >70% preterm formula, and 15 mixed 50% own mother’s milk and preterm formula. Exclusion criteria: congenital infections, congenital malformations and newborns of drug addicted mothers. Stools were collected weekly during the first 28 days. Microbial DNA extraction, 16S rRNA amplification and sequencing were performed.

**Results:**

All groups were similar in perinatal and neonatal data. There were significant differences in microbial community among treatments. Approximately 37% of the variation in distance between microbial communities was explained by use of exclusive own mother´s milk only compared to other diets. The diet composed by exclusive own mother´s milk allowed for greater microbial richness (average of 85 OTUs) while diets based on preferably formula, exclusive formula, preferably maternal milk, and mixed of formula and maternal milk presented an average of 9, 29, 23, and 25 OTUs respectively. The mean proportion of the genus *Escherichia and Clostridium* was always greater in those containing formula than in the those with maternal milk only.

**Conclusions:**

Fecal microbiota in the neonatal period of preterm infants fed with exclusive own mother’s milk presented increased richness and differences in microbial composition from those fed with different proportions of formula.

## Introduction

The intestinal microbiota is very important for human metabolism, development and behavior [1,2]. Despite several studies on the subject and its connection with high complexity diseases [1,3,4], the studies were based on culture, genetic profile and/or the use of small sample sizes, which makes it clear that the variables responsible for shaping the intestinal microbiota have not been satisfactorily examined [1,5,6]. It is known that the development of infant microbiota depends on medical and dietary factors [1,7], but it is not yet known how such factors influence the microbial overall composition and their associations with the human body [1]. The human body has millions of microorganisms that work in partnership with our own cells to influence the quality of our lifelong health [8,9]. The composition of the childhood intestinal microbiome is influenced by factors such as the type of birth, gestational and postnatal age, ingestion of antibiotics, environment, nutritional exposures, and breastfeeding, which should be emphasized as an important variable for the assembly of the intestinal microbiota [5,8,10,11,12]. La Rosa et al have shown that the gut microbioma of premature infants admitted to Neonatal Intensive Care Units progresses and bacterial population changes in composition along the time [6]. Despite the influence of breast milk versus formula in the assembly of the gut microbiota, the true impact of own mother’s breast milk on the composition of the intestinal microbiome of premature infants is not fully understood [8].

The immature intestinal microbiota of premature newborns is influenced by factors such as postnatal age, gestational age, birth weight and nutritional exposures [8,13,14,15]. In the specific case of breastfeeding, this seems to disguise the influence of birth weight, which suggests a protective function against the intestinal immaturity of the premature newborn at the onset of life [8]. These findings suggest not only the existence of a microbial mechanism underlying the body of evidence that elucidates that breast milk promotes the intestinal health of the premature newborn [16], but also the dynamic interaction of host and dietary factors that help in the colonization and enrichment of specific microbes during the establishment of its intestinal microbiota [8].

Therefore, the aim of this study is to describe the intestinal microbiota of very low birth weight infants in the first 28 days depending on use of mother’s own milk or use of formula in different proportions.

## Material and methods

This study used a convenience sampling strategy with patients recruited from the Neonatology Section of Hospital de Clínicas de Porto Alegre (HCPA), Brazil from May 2014 to January 2017. Pregnant women with gestation age ≤ 32 weeks that provided written informed consent were enrolled at hospital admission for their delivery. The study protocol was approved by the Ethics Committee of Hospital de Clínicas de Porto Alegre (HCPA), and the guardians signed an informed consent form. Exclusion criteria were: 1) HIV or congenital infections, 2) mothers with substance abuse and 3) neonatal congenital malformations. Infants´ weekly stool samples were collected from diapers beginning with the first stool until the 4^th^ week of life. All samples were immediately stored in liquid nitrogen until DNA extraction.

Newborns were divided in five groups according to the feeding: exclusive own mother’s milk (LME), exclusive formula (PFL), mixed 50% own mother’s milk and 50% formula (MFLM), mixed with formula and 70% or more of own mother’s milk (PLM), and mixed with own mother´s milk and 70% or more of formula (PFL). The newborns received daily the same diet for up to 28 days. The different amounts of formula and own mother´s milk was offered separately, at different times of the day, so that at the end of the day the ratio was maintained. The ratio of breast milk to formula was the limitation of the amount of breast milk available. The preterm formula used was Pre Nan. This formula does not contain neither probiotics nor prebiotics.

Obstetrical data and neonatal data were collected prospectively.

### DNA extraction and the 16S rRNA library preparation

The laboratory technique as described in detail previously [16].

Microbial DNA was isolated from samples using the QIAmp Fast DNA Stool Mini Kit (Qiagen, Valencia, CA, USA) following manufacturer’s instructions with modifications. All DNA samples were kept at -80°C until use. The V4 region of the 16S rRNA gene was amplified and sequenced using the PGM Ion Torrent platform (Thermo Fisher Scientific, Waltham, MA, USA) with the barcoded bacterial/archaeal primers 515F and 806R [17]. PCR amplification was carried out using barcoded primers linked with the Ion adapter “A” sequence (5’-CCATCTCATCCCTGCGTGTCTCCGACTCAG-3’) and Ion adapter P1 sequence (5’-CCTCTCTATGGGCAGTCGGTGAT-3’) to obtain a sequence of primer composed for A-barcode-806R and P1-515F adapter and primers. Each of the 25μL of PCR mixture consisted of 2U of Platinum Taq DNA High Fidelity Polymerase (Invitrogen, Carlsbad, CA, USA), 4μL 10X High Fidelity PCR Buffer, 2 mM MgSO4, 0.2 mM dNTP's, 0.1 μM both the 806R barcoded primer and the 515F primer, 25μg of Ultrapure BSA (Invitrogen, Carlsbad, CA, USA) and approximately 50 ng of DNA template. PCR conditions used were: 95°C for 5 min, 35 cycles of denaturation at 94°C for 45s; annealing at 56°C for 45s and extension at 72°C for 1 min; followed by a final extension step at 72°C for 10 min.

The resulting PCR products were purified with the Agencourt AMPure XP Reagent (Beckman Coulter, Brea, CA, USA) and the final concentration of the PCR product was quantified by using the Qubit Fluorometer kit (Invitrogen, Carlsbad, CA, USA) following manufacturer's recommendations. Finally, the reactions were combined in equimolar concentrations to create a mixture composed by 16S gene amplified fragments of each sample. This composite sample was used for library preparation with Ion OneTouch 2 System with the Ion PGM Template OT2 400 Kit Template (Thermo Fisher Scientific, Waltham, MA, USA). The sequencing was performed using Ion PGM Sequencing 400 on Ion PGM System using Ion 318 Chip v2 with a maximum of 40 samples per microchip.

### Sequence processing

The 16S rRNA raw sequences were analyzed following the recommendations of the Brazilian Microbiome Project [18]. Briefly, the OTU (Operational Taxonomic Unit) table was built using the UPARSE pipeline [19], in which the reads were truncated at 200 bp and quality filtered using a maximum expected error of 0.5. Filtered reads were dereplicated and singletons were removed. After clustering sequences into OTUs, with a similarity cutoff of 97%, chimeras were checked to obtain representative sequences for each microbial phylotype. Taxonomic classification was carried out using SINTAX [20] against the Ribosomal Database Project (RDP) database [21] with a confidence threshold of 80%. Sampling effort was estimated using Good's coverage [22].

#### Statistical analysis

All clinical data was analyzed using the software SPSS 21.0 at the significance level of 5%. Quantitative variables with normal distribution were described through means/SD and compared by ANOVA with Tukey test. Quantitative variables with asymmetric distribution were described through median/interquartile range and compared by the Kruskall-Wallis test with Dunn Test. For comparison among proportions Pearson's chi-square test was used in conjunction with residue analysis adjusted.

For all 16S rRNA downstream analysis, the data set was filtered, keeping only OTUs that were present in at least 30% of the samples in each treatment and rarefied to the same number of sequences [23]. The biom file was imported into R environment [24] and estimations of alpha and beta diversity were calculated using the “phyloseq” packge [25], and further plotted using the “ggpltot2” package.

To accesses the main differences among treatments in this study, a Permutational Multivariate Analysis of Variance (PERMANOVA) [26] as performed using a binomial dissimilarity matrix among samples and the *adonis* function implemented in the vegan package [27].

Differences between treatments were accessed through the STAMP software [28]. P-values were obtained by the two sided White’s non-parametric t-test followed by the Benjamini-Hochberg FDR correction. A p-value < 0.05 together with effect size filter (difference between proportions effect size <1.00) was applied to determine the most important taxa that differed between treatments.

Analysis along the 28-day period was performed using meta-analysis of effect sizes reported at multiple points using general linear mixed mode [29].

## Results

A total of 175 samples from 62 preterm newborns divided in five groups (7 in LME, 8 in FLE, 16 in PLM, 16 in PFL, and 15 in MFLM) were collected and analyzed. The five groups were similar in respect to maternal and obstetrical data (Table 1).

**Table 1:** Characteristics of the subjects.

Variables	Exclusive breast milk (n = 7)	Exclusive Formula (n = 8)	Predominance of breast milk (n = 16)	Predominance of formula (n = 16)	Mixed (n = 15)	p-value
Maternal age (years)—mean ± SD	24.0 ± 9.1	26.1 ± 6.4	31.6 ± 5.5	24.6 ± 7.4	28.0 ± 7.5	0.052
C-section–n(%)	7 (100)	6 (75)	13 (81.3)	11 (68.8)	12 (80)	0.556
Rupture of membranes (hours)–median (P25-P75)	0 (0–3)	0 (0–0.4)	0.04 (0–37)	6.1 (0–96.5)	0 (0–3)	0.246
Rupture of membranes ≥18 hours–n(%)	0 (0.0)	1 (12.5)	4 (25)	7 (43.8)	2 (13.3)	0.062
Maternal antibiotic–n(%)	3 (42.9)	5 (62.5)	11 (68.8)	14 (87.5)	8 (53.3)	0.188
Preeclampsia–n(%)						0.119
Yes	5 (71.4)	1 (12.5)	7 (43.8)	6 (37.5)	4 (26.7)	
No	2 (28.6)	6 (75)	9 (56.3)	10 (62.5)	11 (73.0)	
Eclampsia	0 (0.0)	1 (12.5)	0 (0.0)	0 (0.0)	0 (0.0)	
Streptococcus–n(%)	1 (14.3)	2 (25)	4 (25)	5 (31.3)	6 (40)	0.944

Statistically significant association by the test of the residuals adjusted to 5% of significance

The mean, the median standard deviation and the interquartile range were used, using Analysis of Variance (ANOVA) in conjunction with the Tukey test was applied. In case of asymmetry, the Kruskall-Wallis test in set with Dunn were used. Categorical variables were described by absolute and relative frequencies. In the comparison of proportions, Pearson's chi-square test was used in conjunction with residue analysis adjusted

Neonatal data are shown in Table 2.

**Table 2:** Neonatal data according to study group.

Variables	Exclusive breast milk (n = 7)	Exclusive Formula (n = 8)	Predominance of breast milk (n = 16)	Predominance of formula (n = 16)	Mixed (n = 15)	p-value
Birth Weight (g)—mean ± SD	912.1 ± 291.5^a^	1684 ± 430.1^b^	1460 ± 575.4^ab^	1459 ± 413.3^ab^	1332 ± 372^ab^	0.021
Gestational age (weeks)—mean ± SD	27.7 ± 2.7^a^	30.6 ± 1.7^b^	29.9 ± 2.4^ab^	30.5 ± 1.6^b^	29.4 ± 1.7^ab^	0.031
NEC-n (%)	1 (14.3)	0 (0.0)	1 (6.3)	1 (6.3)	2 (13.3)	0.778
Use of antenatal corticosteroid—n (%)	6 (85.7)	7 (87.5)	16 (100)	15 (93.8)	15 (100)	0.185
Early onset sepsis—n (%)	0 (0.0)	0 (0.0)	0 (0.0)	0 (0.0)	1 (6.7)	0.527
Use of Antibiotics on the first week—n (%)	6 (85.7)	3 (37.5)	12 (75)	13 (81.3)	8 (53.3)	0.110
Late onset sepsis—n (%)	2 (28.6)	3 (37.5)	7 (43.8)	5 (31.3)	6 (40.0)	0.937
Use of Antibiotics on the 2nd week—n (%)	7 (100)	5 (62.5)	10 (62.5)	10 (62.5)	41 (66.1)	0.396

Statistically significant association by the test of the residuals adjusted to 5% of significance

For the Weight at birth and Gestational age the mean and standard deviation were used. We compare the averages through Analysis of Variance (ANOVA) in conjunction with the Tukey test. In case of asymmetry the Kruskall-Wallis test in conjunction with Dunn were used.

Categorical variables were described by absolute and relative frequencies. In the comparison of proportions, Pearson's chi-square test was used in conjunction with residue analysis adjusted.

### Overall microbial community differences among diets

**Fig 1:**
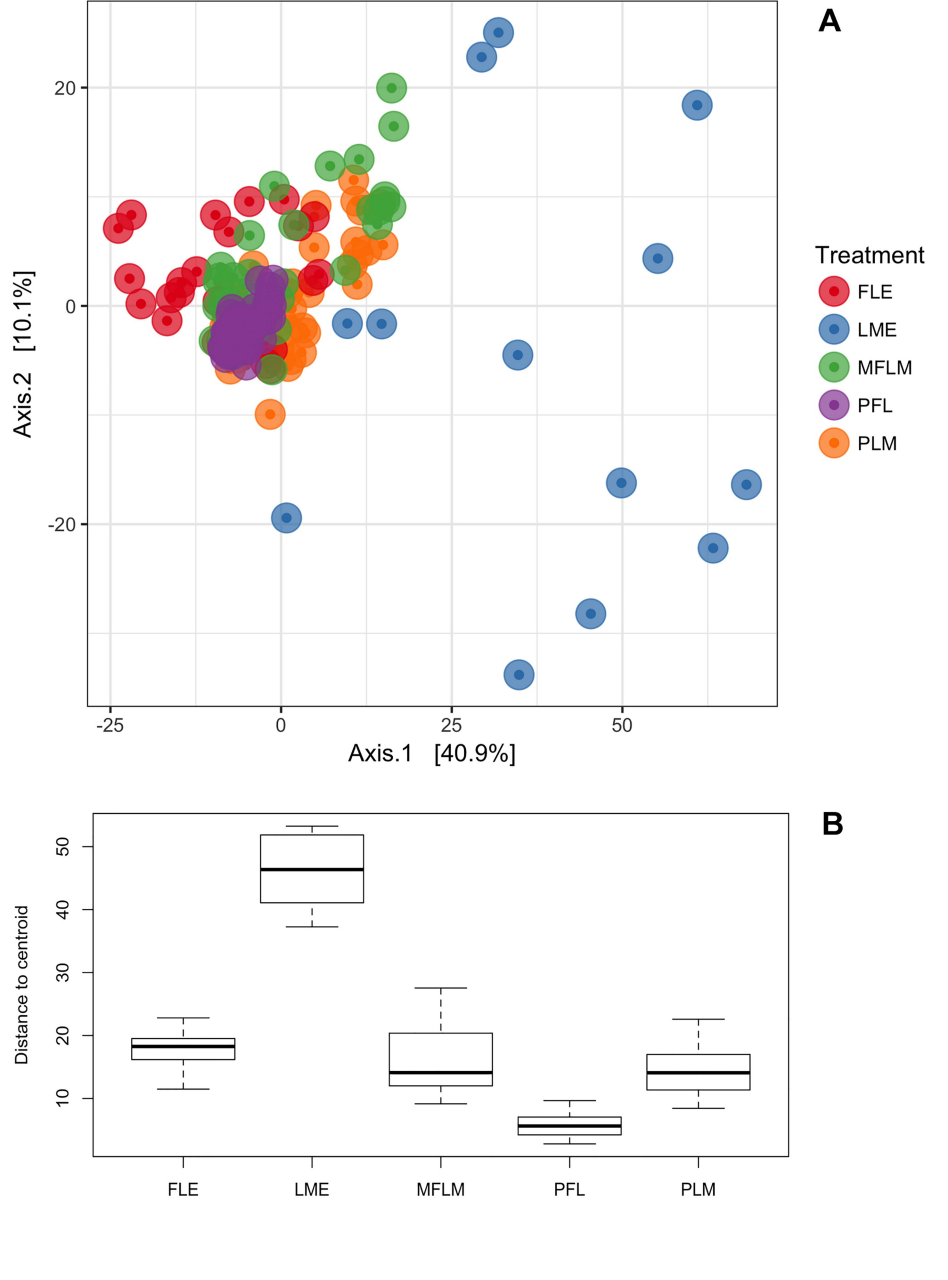
Beta diversity comparisons among microbial communities. **A and B.** Graph A represents clusters of microbial communities. Each point represents an individual sample, with colors indicating feeding treatments. Graph B represents measurement of multivariate dispersion for each treatment. FLE = exclusive formula; LME = exclusive own mother´s milk; MFLM = 50% formula and 50% own mother’s milk; PFL = ≥70% formula; PLM = ≥70% own mother’s milk.

The microbial community differences at OTU level (97% similarity cutoff for grouping definition) found among fecal samples from preterm newborns’ fed with five different diets during a period of 28 days are presented in Fig 1. The ordination analysis revealed significant differences in microbial community structure among treatments suggesting the feeding of different diets was responsible for the assembly of preterm gut community. Those differences were further confirmed by the permutational multivariate analysis of variance (Table 3).

**Table 3:** Permutational multivariate analysis of variance comparing microbial communities found in fecal samples from preterm newborns fed with different diets during 28 days.

	F	R^2^	Adjusted p-value
**All samples**			
Diets	19.24	0.311	0.001
Residuals		0.688	
Total		1.000	
**Pairwise comparisons**			
PLM *vs* FLE	14.17	0.168	0.01
PLM *vs* LME	21.89	0.274	0.01
PLM *vs* MFLM	7.28	0.080	0.01
PLM *vs* PFL	16.14	0.146	0.01
FLE *vs* LME	18.85	0.331	0.01
FLE *vs* MFLM	10.82	0.146	0.01
FLE *vs* PFL	12.92	0.148	0.01
LME *vs* MFLM	18.88	0.270	0.01
LME *vs* PFL	36.76	0.372	0.01
MFLM *vs* PFL	14.78	0.145	0.01

F = F value by permutation. R^2^ = shows the percentage of variation explained by diets; p-values were based on 999 permutations and were adjusted by Bonferroni correction.

FLE = exclusive formula; LME = exclusive own mother´s milk; MFLM = 50%formula and 50% own mother´s milk; PFL = ≥70% formula; PLM = ≥70% own mother´s milk.

The overall analyses (all samples) indicate that approximately 31% of the variation in distances among treatments was explained by the different diets provided in each treatment. Pairwise comparisons revealed that diets based on maternal milk assembled microbial communities with large variation within group (e.g. greater differences among microbial communities from subjects feed with maternal milk) while diets based on formula created more similar microbial communities (Fig 1A and 1B) (Table 3). The use of maternal milk was responsible for the greatest variation observed between diets. The highest variation between treatments was observed among the samples under exclusive own mother’s milk and samples under exclusive formula. Approximately 37% of the variation in distance between microbial communities could be explained by the treatment with maternal milk only (LME) compared to the diet based preferentially in formula (PFL).

Besides, the greatest variations in distances between treatments were observed in those comparisons involving the use maternal milk only. Approximately 37% of the variation in distance between microbial communities could be explained by the treatment with maternal milk only compared to the diet based preferentially in formula (Table 3).

**Fig 2:**
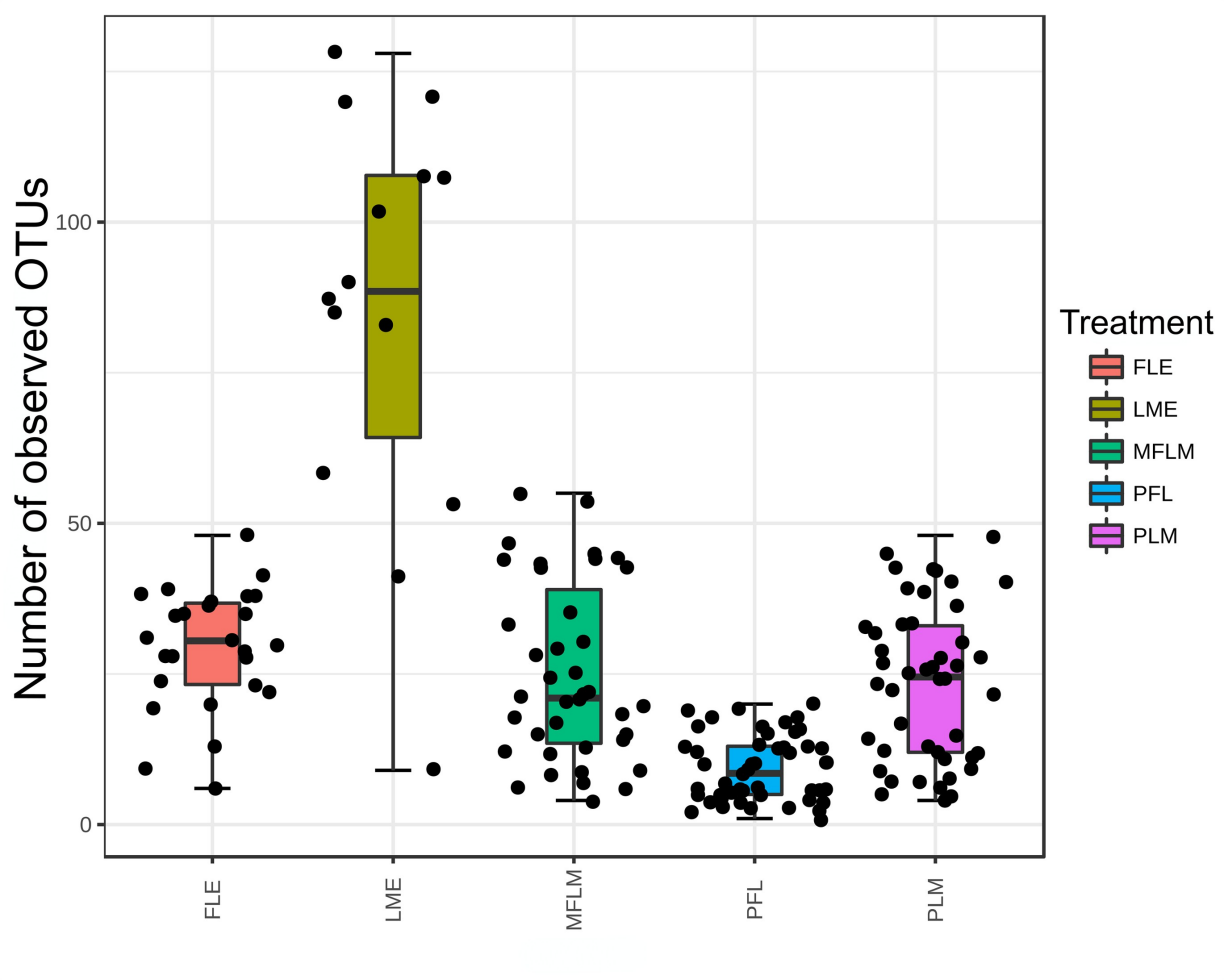
Number of Operational Taxonomic Units measured in fecal samples from preterm babies fed with different diets during 28 days. Boxes span the first to third quartiles; the horizontal line inside the boxes represents the median. Whiskers extending vertically from the boxes indicate variability outside the upper and lower quartiles. Treatments were significant different according to the Kruskal-Wallis test (p-value < 0.001), with greatest diversity in human milk exclusive group. FLE = exclusive formula; LME = exclusive own mother’s milk; MFLM = 50%formula and 50% own mother’s milk; PFL = ≥70% formula; PLM = ≥70% own mother’s milk. All libraries were rarefied at the same number of sequences prior to OTUs analysis.

In agreement with the ordination analysis, alpha diversity measurements indicated significant differences (p-value < 0.001 according to the Kruskal-Wallis test) among the richness of OTUs within treatments (Fig 2). The diet composed by maternal milk only (LME) allowed for greater microbial richness (average of 85 OTUs). On the other hand, the diet preferably based in formula (PFL) presented the smallest richness (average of 9 OTUs) (Fig 2). The average number of OTUs found within the other diets was similar. The diets based in formula only (FLE) and preferably maternal milk (PLM) presented an average of 29 and 23 OTUs respectively and the diet based in a mixture of formula and maternal milk presented an average of 25 OTUs.

### Identification of the main taxonomic differences among diets

**Fig 3:**
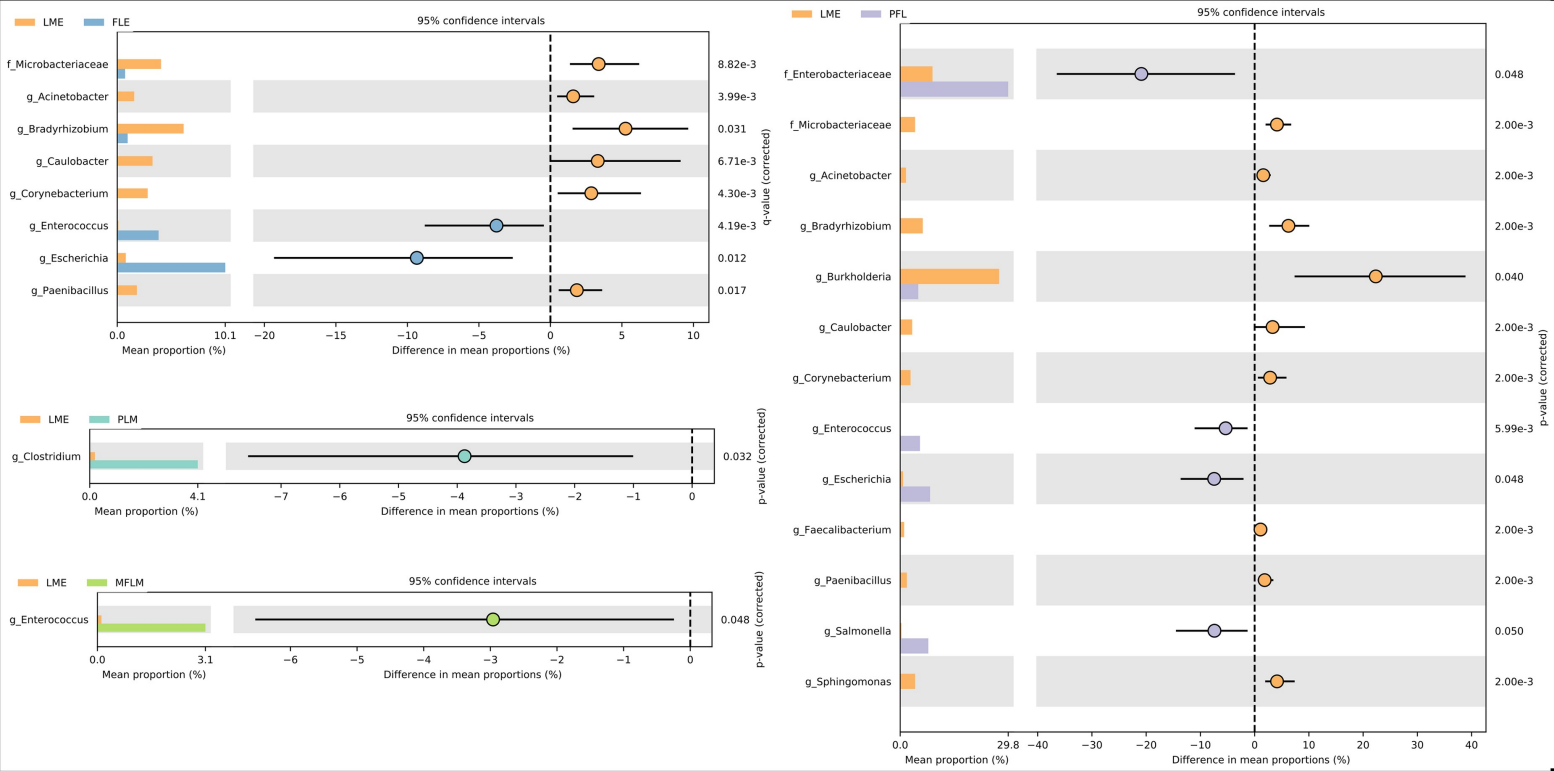
Differential microbial abundance among microbial communities detected in samples from preterm babies fed with different diets during 28 days. p-values were obtained by the two-sided White’s non-parametric t-test followed by the Benjamini-Hochberg FDR correction. A p-value of < 0.05 together with effect size filter (difference between proportions effect size <1.00) was applied to determine the most important taxa that differed between treatments. Only statistically significant differences are shown.

Once overall differences among microbial communities found in fecal samples from preterm newborns fed with different diets during 28 days were detected, the next step was to identify the microbial taxa responsible for that difference. To detect differences among treatments at genus level, a pairwise differential abundance based on a two-sided White’s non-parametric t-test followed by the Benjamini-Hochberg FDR correction was performed. As LME treatment presented the greatest difference in microbial community among diets, the pairwise comparisons were performed between LME and the other diets (Fig 3).

The mean proportion of the genus *Escherichia* was always greater in treatments containing formula (FLE, PLM, MFLM and PFL) than in the treatment with maternal milk only. Particularly, the diet based on maternal milk presented an increased abundance of *Acinetobacter*, *Bradyrhizobium*, *Caulobacter*, *Corynebacterium* and *Paenibacillus*, *Burkholderia*, *Faecalibacterium*, *Sphingomonas* and the unknown genus from the *Microbacteriaceae* family as compared with the other treatments. Compared to the diet based on maternal milk, the diet preferably based on maternal milk (PLM) and the diet composed by a mixture of formula and maternal milk increased the abundance of *Clostridium* and *Escherichia*. The fecal samples from newborns fed with a diet preferable based on formula (PFL) presented greater abundance of *Escherichia*, *Salmonella*, *Enterococcus* and the unknown genus from the *Enterobacteriaceae* family when compared to the LME treatment (Fig 3).

## Discussion

The variables influencing the composition of the intestinal microbiota are topic of multiple studies. The assumption is that, once they are known and understood, new strategies can be developed to maintain a state of health [30,31,32]. In this study, we found global differences in the microbial community among the types of milk administered to preterm infants, showing that the greatest microbial richness was found in those who were exclusively fed with own mother´s milk. Approximately 37% of the variation in the distance between microbial communities was explained by treatment with breast milk exclusively, in comparison with diets based preferably on formula.

Knowing the total number of bacteria according to type of feeding allow us an understanding of certain situations which facilitate the development of diseases [8,31,32,33]. In our study, fecal samples of premature infants fed different diets were decisive in the diversity of the microbial community during the 28-day period. All infants were premature and were separated according to the type of diet fed. Feeding them own mother´s milk exclusively allowed for greater microbial richness (mean of 85 OTUs). The formula-based group had the lowest richness (mean of 9 OTUs). These diets based on the exclusive offer of formula and, preferably, breast milk, showed an average of 29 and 23 OTUs respectively; and the diet based on a mixture of formula and breast milk presented an average of 25 OTUs.

Gregory et al studied 30 preterm infants (10 in each group) during the first 60 days of life fed with maternal breast milk, pasteurized donor human milk and preterm infant formula. It was found that those fed with maternal breast milk presented higher diversity [8]. Cacho et al showed that, in vitro, incubation of own mother´s milk with donor breast milk in a certain percentage and for 4 hours, may restore the maternal milk microbiome [31]. We did not mix human milk with formula. We offered them individually in different proportions throughout the day. Our data showed that this unmixed administration of breast milk and formula does not determine the restoration of human milk microbioma.

In our study the average proportion of the *Escherichia* and *Clostridium* genus were always higher in treatments containing formula than in treatment which provided breast milk exclusively. This finding has been reported previously [6]. A possible explanation for the results is found in the transmission carried out from the mother to the child and the external environment after the birth, as has already been indicated in other studies [33,34]. Besides that, there are factors like lactoferrin and glycoproteins in human milk that are protective against pathogen bacteria [35, 36].

In spite of several studies on the topic and the subject’s relation with diseases of high complexity [1,3,4], such studies were restricted to the enumeration of such diseases based on culture, genetic profile (16S) and the use of small samples, which makes it clear that the variables that shape the intestinal microbiota have not been satisfactorily examined [1,5,6]. It is a known fact that the development of the microbiota in infants depends on medical and dietary factors (1,7), but it is not yet known how such factors influence the general composition of the microbiota, and how those factors cooperate with each other [1]. Studies based on fecal samples of infants and their mothers contribute for monitoring of each chronological and functional stage during the first year of life [1,34].

Besides contributing to the microbial richness, breast milk also favors the prevention of sepsis, necrotizing enterocolitis (NEC) and other diseases [29,31,33,37]. NEC is one of the main causes of morbidity and mortality in neonatal intensive care units, with most cases occurring among premature infants [32,37]. In another study of our group, we provide evidence of an association between NEC and distortions in the normal development of the microbiota and low diversity in NEC cases [32]. Within this study we found that increased diversity and breast feeding correlate with reduced incidence of NEC [32]. Mai et al reported a microbiota that predisposed to late onset sepsis in preterm infants that was closer to that we found in preterm newborns that were not fed exclusively with own mother´s breast milk [38].

Other studies emphasize the influence of diet according to the ethnic and/or geographic characteristics as variables that influence the development of newborn intestinal microbiota [39,40,41]. In our study, these issues were not addressed because they were not the focus of the study, leaving open perspectives for innovative studies.

The microbiome found in breast milk contributes in the short and long term to the prevention of colonization by pathogens, as it stimulates the production of reactive antibodies and establishes a healthy intestinal microbiome capable of preventing long-term morbidities such as obesity, type 2 diabetes, chronic intestinal inflammation, autoimmune disorders, allergies, irritable bowel syndrome and allergic gastroenteritis [31,42,43,44]. Our study confirms that the intestinal microbiota of preterm infants presents differences according to their diet—whether breast milk or formula—and emphasizes the importance of breast milk in the maintenance of microbial richness of the newborn's microbiota. *Escherichia* and *Clostridium* have been associated to NEC as well as a high proportion of *Proteobacteria* with few numbers with *Firmicutes* [45].

Some situations, however, crossed this research, such as the loss of fecal samples; the difficulty of mothers in breastfeeding preterm infants with breast milk exclusively due to socioeconomic difficulties, for example; and the low fecal volume produced by premature infants. Another important point to consider when analyzing the intestinal microbiota of the newborn, we analyze only the stools and we do not know the microbiota of the proximal colon neither of the small bowel. We could not adjust microbial analysis for birth weight and gestational age because the number of subjects in exclusive breast milk group was not big enough. It is very difficult to have extreme premature newborns fed exclusively with breast milk during the whole period in NICU hence we were not allowed to study different variables. Our objective was to compare exclusive own mother´s milk feeding with different proportion of formula feeding. We studied just extreme premature newborns, all of them included in the same category of prematurity, and those small differences in birth weight and gestational age probably do not have major repercussion on microbioma.

Many studies have focused on the issue of breastfeeding, particularly regarding the newborns' microbiota, to identify its benefits for the development and prevention of diseases throughout their lives. Considering the importance of the topic, this study aimed to describe the intestinal microbiota of preterm newborns according to their nutritional habits establishing modifications of the intestinal microbiota according to the type of enteral diet administered.

Based on our data, it is noticed that global differences of the microbial community are found among the types of diets administered to preterm infants, showing that the greatest microbial richness was found in those who received exclusive own mother’s milk in comparison with those that received different proportion of formula.
